# Validation of the American English Acute Cystitis Symptom Score

**DOI:** 10.3390/antibiotics9120929

**Published:** 2020-12-19

**Authors:** Jakhongir F. Alidjanov, Kurt G. Naber, Adrian Pilatz, Florian M. Wagenlehner

**Affiliations:** 1 Clinic for Urology, Pediatric Urology and Andrology, Justus Liebig University, Rudolf-Buchheim-Str. 7, 35392 Giessen, Germany; adrian.pilatz@chiru.med.uni-giessen.de (A.P.); Florian.Wagenlehner@chiru.med.uni-giessen.de (F.M.W.); 2 Department of Urology, Technical University of Munich, 81675 Munich, Germany; kurt@nabers.de

**Keywords:** acute cystitis symptom score, ACSS, cystitis, urinary tract infection, female patients, diagnosis, patient-reported outcome

## Abstract

The diagnosis of acute uncomplicated cystitis (UC) is usually based on clinical symptoms. The study aims to develop and validate the American-English Acute Cystitis Symptom Score (ACSS), a self-reporting questionnaire for diagnosis and patient-reported outcome in women with acute uncomplicated cystitis (UC). After certified translation into American-English and cognitive assessment, the clinical validation of the ACSS was performed embedded in a US phase-II trial. 167 female patients with typical symptoms of UC were included in the study following US Food and Drug Administration (FDA) guidance. At Day 1 (diagnosis), the mean (SD) sum score of the six ACSS typical symptoms reached 10.60 (2.51). Of 100 patients followed-up last time on Day 5 or 6 (End-of-treatment, EoT), 91 patients showed clinical success according to the favored ACSS criteria (sum score of typical symptoms 0.98 (1.94)). There was no correlation between the severity of symptoms on Day 1 or between clinical success rate at EoT and level of bacteriuria on Day 1. The American-English ACSS showed high predictive ability and responsiveness and excellent levels of reliability and validity. It can now be recommended as the new master version in clinical and epidemiological studies, in clinical practice, or for self-diagnosis of women with symptoms of UC.

## 1. Introduction

Acute cystitis is the most frequent bacterial infection in women [1]. The diagnosis of acute uncomplicated cystitis (UC) can be made with high probability based on a focused history of lower urinary tract symptoms and the absence of vaginal discharge or irritation [2]. Various urinary symptoms have been used to assess the diagnosis and severity of UC in women [3,4,5,6,7], but only a few studies developed a questionnaire to also evaluate the severity and impact on activity impairment [5,6], which, however, were not designed for diagnostics of UC, but only for follow up.

The Acute Cystitis Symptom Score (ACSS) is a simple and self-reporting questionnaire for female patients with UC, allowing not only assessment of the presence but also the severity of typical and differential symptoms, quality of life, as well as considering additional health conditions and possible changes after therapy [8,9,10]. The ACSS has proven to be a valuable instrument for clinical studies and medical practice for initial diagnosis, as well as a patient-reported outcome (PRO) measure allowing the monitoring of the efficacy of therapy in women suffering from UC [8,9,10]. According to the US Food and Drug Administration (FDA) guidance [11] and European Medical Agency (EMA) draft guidelines [12], the clinical response is important not only for the primary composite efficacy endpoint, but also at each fixed time point assessment as a secondary endpoint, meaning that the ACSS could also be used as a well-defined PRO measure instrument. The current study aimed to develop and validate the American English version of the Acute Cystitis Symptom Score (ACSS).

## 2. Results

### 2.1. Linguistic Validation

**Figure 1:**
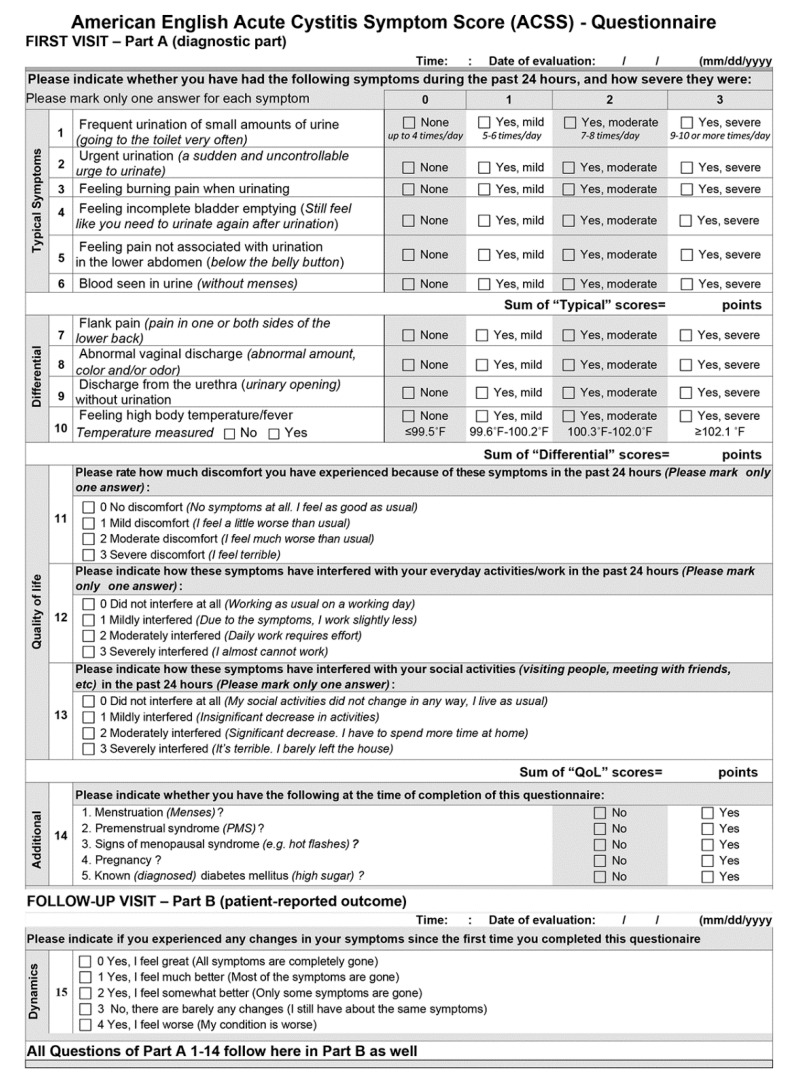
American English Acute Cystitis Symptom Score (ACSS)—Questionnaire.

After the certified translation from the source language, Russian, into the target language, American English, the ACSS was discussed further and adapted slightly by the Scientific Committee (SC), with the consideration of the FDA recommendations [13]. The cognitive assessment was performed by 10 US physicians and 49 females aged 19–87 years, with American English as their first language, of different races (white 81.6%), of different educational levels (Grade School 2.0%; High School 30.6%; College 55.1%; Postgraduate 12.2%), of different reasons for their doctor visit, and with history (73.5%) or without history (26.5%) of UC [13]. Feedback from these females and physicians were discussed within the SC, and after necessary corrections and an appropriate update, the final version of the ACSS (Figure 1) was established and used in the US Phase II trial as mentioned above.

### 2.2. Study Population of Clinical Validation

A total of 167 female patients aged 30 (17–87) median (range) years with typical symptoms of AC at 11 sites were included in the clinical study, which followed the FDA guidance [11]. Demographic characteristics of the US and the international cohorts are provided in Table 1 with a mean (SD) age of 36.8 (15.3) and 34.6 (15.1) years, respectively. Cohorts were homogenous concerning age or additional conditions except for pregnancy because pregnant women were not included in the US study according to FDA guidance [11].

### 2.3. The ACSS in US Cohort at Day 1 (Diagnostics)

Of the 167 patients, 162 (97.0%) achieved at Day 1 a sum score of the “Typical“ domain (ACSS) of 6 and higher, which shows an excellent agreement between the clinical diagnosis made by the treating physician according to FDA guidance [11] and the patient’s symptoms scoring using the ACSS questionnaire [8,9].

Detailed questionnaire data for the US cohort on Day 1, including the severity of symptoms and impact on the quality of life (QoL) are provided in Table 2. About 80–90% of patients complained of the moderate-to-severe intensity of the following symptoms: urinary frequency, urinary urgency, dysuria, and sense of incomplete bladder emptying.

Test of the internal consistency of the items of the ACSS resulted in Cronbach’s alpha (95% CI) of 0.89 (0.87; 0.91) for “Typical” domain (r = 0.58), 0.46 (0.35; 0.56) for the “Differential” domain (r = 0.15), 0.96 (0.95; 0.97) for the “QoL” domain (r = 0.88), and 0.90 (0.88; 0.92) for the total questionnaire (r = 0.39).

### 2.4. Comparison of the ACSS between the US and International Cohort at Day 1 (Diagnostics)

**Figure 2:**
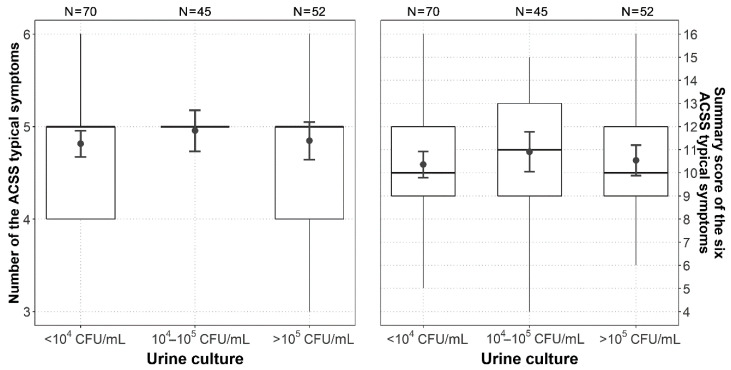
Boxplots (IQR, range, mean ± SD) of the number present and sumscore of the six ACSS typical symptoms in the US group versus the three categories of bacteriuria at Day 1.

The comparison between the US (167) and the international cohort (237), in which the ACSS was used in the following languages [9]: Uzbek (313), Russian (87), Tajik (58), German (43), and Hungarian (16), did not show any statistically significant difference in the average number and sum scores of the “Typical” and “Quality of Life” domains, as well as in the sum score of the entire ACSS. The sum score of the “Differential” domain (such items as flank pain, vaginal and urethral discharge, and elevated body temperature) was lower for the US cohort compared to the international cohort and the difference was statistically significant (*p* = 0.003) (Table 3, Figure S1 and Figure S2). There was no significant association between the amount and/or severity of symptoms on Day 1 and the level of bacteriuria on Day 1 (Figure 2).

### 2.5. Patient-Reported Outcome Before, During and After Therapy

**Figure 3:**
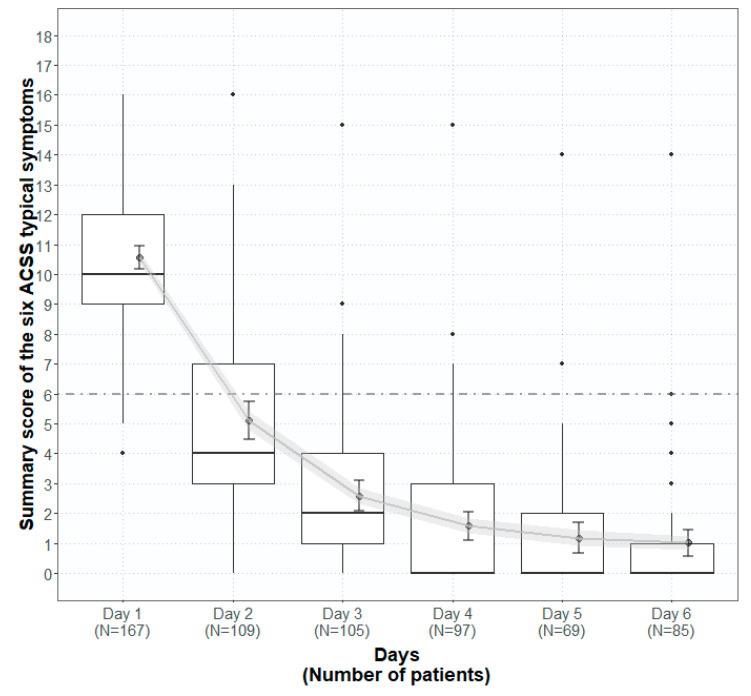
Boxplots (IQR, range, mean ± SD, error of mean) of the sumscore of the six ACSS typical symptoms in the US group on Day 1–6.

Figure 3 represents the summary score of the typical domain of the ACSS on Day 1–6 of the US cohort. The results demonstrated that after high sum scores at Day 1 before treatment (mean 10.6), for the following days during treatment, the sum scores were reduced quite distinctly and reached almost a plateau on Days 5 and 6. Therefore we investigated more carefully the 100 patients who filled in the ACSS questionnaire on Day 1 and the last time either on Days 5 or 6.

Table 4 demonstrates the sum scores of typical symptoms (total and concerning the amount of bacteriuria at Day 1), differential symptoms, and quality of life of the patients in the US cohort at Day 1 (Baseline) and Days 5/6 (EoT). The reduction from Day 1 to end-of-treatment at Days 5/6 was highly significant for all ACSS domains.

Correlation test for the sum scores of the “Typical“ domain stratified according to the amount of bacteriuria at Day 1, has proven our hypothesis about the absence of significant relationships between the severity of UC or clinical outcome at EoT and the amount of bacteriuria at the time of the start of the therapy (Table 4).

Table 5 shows in more detail the severity of the individual typical and differential symptoms, their impact on each of the quality of life categories, and the overall patient’s assessment of symptomatic changes (ACSS “Dynamics“) at EoT claimed by the same 100 patients of the US study cohort at Day 1 and 5/6 (EoT)

### 2.6. Comparison of Patient-Reported Outcome Between the US and the International Cohort

Table 6 shows the number of cases when using certain breakpoints to determine success and non-success at Days 5/6 in the patients of the US (n = 100) and international study cohort at Day 5–9 (EoT) (n = 82) [10]. Of the five predefined thresholds, the threshold A (Sum score of typical domain ≤5 scores, no item >1 and “visible blood in urine” = 0) and D (Sum score of 4 FDA symptoms ≤4, no item >1 and “visible blood in urine” = 0) are favored and showed the same results with a success rate of 91% in the US and 80% in the international cohort. The higher success rate in the US cohort can probably be explained because all patients were treated with a suitable antibiotic, whereas the non-interventional treatment of the international cohort might have varied considerably.

### 2.7. Validation of the American English ACSS Using Pre- and Posttreatment Results

The diagnostic values of the different items and the sum score of the “Typical“ domain of the ACSS were tested, comparing results obtained by the ACSS before and after treatment.

**Figure 4:**
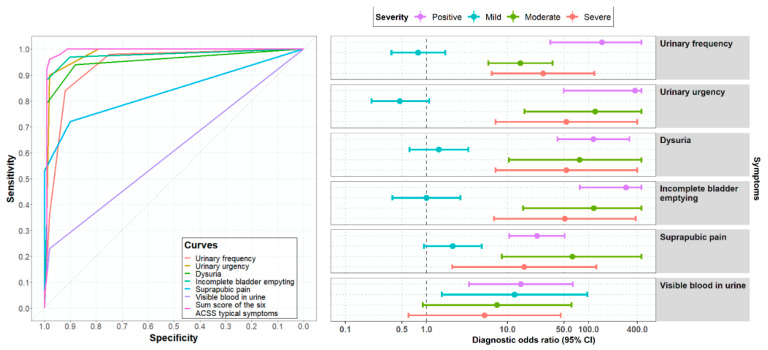
Receiver operating characteristic (ROC) curves and diagnostic odds ratios for the ACSS typical symptoms in 100 patients of the US group comparing the results obtained at Day 1 (diagnostics) and Day 5/6 (end of treatment).

Figure 4 illustrates that not only the pure presence of typical symptoms, but rather severity (scores) increases the diagnostic accuracy as has been shown earlier [7]. Receiver operating characteristic (ROC) curves for each independent item and the sum score of the “Typical” domain demonstrated the best-balanced results for sensitivity (96%, 95% CI: 90–99%) and specificity (98%, 95% CI: 93–100%) for the sum score of 6 typical symptoms as compared to the individual symptoms. More detailed calculations, such as positive and negative predictive values, positive and negative likelihood ratio, and correlation with a positive outcome (diagnosis of UC) comparing different clinical diagnostic definitions are shown in Table 7. Again a sum score of 6 or more of the “Typical“ domain (ACSS) showed the most favorable results as compared, e.g., for the FDA [11] and EMA [12] inclusion criteria for the clinical diagnosis of UC. At a sum score of the “Typical“ domain (ACSS) of 6 the clinical diagnosis of UC can be made best balanced with a sensitivity of 94% and 87% and a specificity of 90% and 88% in two studies including 286 females (139 with UC and 147 without UC) and 517 females (285 with UC and 232 without UC), respectively [8,9]. Therefore, a threshold of a sum score of 6 and higher of the “Typical domain (ACSS) was used for the clinical diagnosis of UC. 

## 3. Discussion

The ACSS, validated in several other languages (www.acss.world), was now also successfully translated and linguistically and clinically validated in American English.

Psychometric parameters and diagnostic values of the American English ACSS showed good-to-excellent results, which are comparable to the Russian as source and other versions as previously translated and validated in different languages [8,9,10,14,15,16,17,18].

Comparing the results obtained by the ACSS for diagnostics of acute UC and EoT showed again that not only the presence but also the severity (scoring) of the symptoms are important, which may be typical but not specific for acute UC because some of the symptoms can also be caused by other urological diseases. It also can be shown that for clinical diagnostics, the best balance between sensitivity and specificity can be obtained using a sum score of 6 or higher of all the six ACSS typical symptoms as has been shown earlier as well [8,9,15].

Using five different reasonable thresholds to differentiate between success and non-success resulted in the same outcome for the US study, but each threshold showed slightly different outcomes in the international group. The study shows again that the ACSS could also be used as a PRO measure instrument in prospective, randomized clinical studies comparing different treatment modalities.

By using the ACSS in this US clinical trial, it could be shown for the first time that the severity of symptoms and the clinical outcome do not correlate with the level of bacteriuria before treatment. There was no difference for both parameters (severity of symptoms and clinical outcome), whether the urine culture showed 10^5^ CFU/mL (as requested by FDA and EMA for microbiological outcome assessment), 10^4^ CFU/mL or <10^4^ CFU/mL.

Since it has been shown in several studies that much lower amounts of bacteriuria are also clinically significant, clinical diagnostics and patient-reported outcome using validated questionnaires should become a priority in women with acute UC, especially when symptomatic treatment modalities are also involved [19,20,21]. Nevertheless, microbiological investigations should not be neglected, but much lower amounts of bacteriuria need to be considered by using appropriate laboratory methods.

## 4. Material and Methods

### 4.1. Study Design

The clinical study was initiated, supported, and designed by Mission Pharmacal Company, San Antonio, TX 78239, USA, as a randomized, double-blind, placebo-controlled, multicenter Phase II trial of the efficacy and safety of MPC-SHRC for the relief of symptoms associated with uncomplicated UTI (ClinicalTrials.gov Identifier: NCT03129295; https://clinicaltrials.gov/ct2/history/NCT03129295?V_1=View) with the ACSS accepted as a study tool. The primary study protocol was approved by the Western Institutional Review Board, 270717. Further details on the study protocol are to be published elsewhere.

### 4.2. The ACSS as a Study Tool

The ACSS is a 2-part, self-reporting questionnaire (Figure 1), including the following questions:

(i). Six questions about typical symptoms of UC (“Typical“ domain): urinary frequency, urinary urgency, dysuria, incomplete bladder emptying, suprapubic pain, visible blood in the urine.
(ii). Four questions regarding differential diagnosis (“Differential“ domain): flank pain, abnormal vaginal discharge, urethral discharge, elevated body temperature/fever.
(iii). Three questions on the quality of life (“QoL“ domain): general discomfort, interference with everyday activity/work, interference with social life.
(iv). All questions of the domains i–iii are to be answered according to severity (scoring 0–3): no (0), mild (1), moderate (2), severe (3).
(v). 5 questions on additional conditions, which may affect therapy (“Additional“ domain): menstruation, premenstrual syndrome, menopause, pregnancy, diabetes mellitus. The answers are yes or no.
(vi). 5 questions on the patient’s assessment of overall symptomatic changes after the baseline visit (“Dynamics“ domain). The answers are rated (scored): Feeling normal (0), much better (1), somewhat better (2), barely any change (3), worse (4).
(vii). Part A includes the domains i-iii, v (Typical, Differential, QoL, Additional) and Part B includes the domains vi (Dynamics) and i-iii, v as in Part A.

### 4.3. Linguistic Validation of the American English Version of the ACSS

The linguistic validation process included two stages: (1) The certified translation into American English from original Russian [14,22,23] by Mapi Language Services, Lyon, France (Ref.No.16-053816) according to international guidelines with forward and backward translations and consideration of comments from subjects interviewed with American English as a first language [24,25,26], and (2) cognitive assessment by 10 US physicians, and by 49 US female subjects with and without a history of UC. The whole process was steered by a scientific committee (SC).

### 4.4. Clinical Validation

The clinical validation was performed as an embedded study of the above-mentioned Phase II trial following the study protocol, Good Clinical Practice, and Code of Federal Regulations Title 1, Parts 50, 56, and 312. The study consisted of two on-site visits: Visit 1 (Day 1) (baseline) and visit 2 (discharge). After the baseline examination, all subjects were randomized to receive either the investigational drug or placebo QID for 3 days. All randomized subjects received concurrent antibiotic treatment as prescribed by the investigator. Before any study-related activities, written informed consent was signed and personally dated by the subject.

The ACSS was first completed on Day 1 (diagnosis) before therapy and was then completed daily until 48 h after the end of treatment (EoT). In this side study, the blinding concerning the investigational drug or placebo was maintained. The scores of the ACSS questionnaire in the US study cohort were compared with those obtained in an international cohort of 237 female patients with acute UC from other studies [10], who have used the ACSS in their native languages (Uzbek, Russian, Tajik, German, and Hungarian), and who also fulfilled the FDA guidance [11] concerning requirements for clinical diagnosis of acute UC with at least two of the following four typical symptoms: urinary frequency, urinary urgency, dysuria, suprapubic pain, and in addition evidence of pyuria in urinalysis. The amount of urine culture (colony forming units/mL) was tested concerning the presence and severity of the ACSS typical symptoms and the clinical outcome at EoT.

### 4.5. Data Acquisition and Processing

The data of the American English ACSS were retrieved from the US cohort. The ACSS data for the international cohort were retrieved from the e-USQOLAT database as described earlier [9,10]. Since US cohort consisted of only the patients, the data at Day 1 (Baseline) of the US cohort were used to define the data of positive outcome/“Patients”, and the last data obtained at Days 5 or 6 (EoT) were used to define negative outcome/“Controls” to assess psychometric reliability and diagnostic value of the American English ACSS. The presence of symptoms (positive, negative), symptoms’ severity (mild, moderate, severe), and the proposed diagnostic approaches (FDA, EMA, ACSS) were considered. Data processing included a procedure of dichotomization of variables for the assessment of diagnostic values. Relative variables were labelled as “0” for “negative / not match”, and “1”-for “positive / match”, as described in detail earlier [9,10].

### 4.6. Statistical Analysis

Variables were visually tested for the normality using Q-Q plots as well as numerically, using Shapiro–Wilk’s test. Descriptive statistics were presented using mean, standard deviations (SD), 95% confidence intervals (CI), median and interquartile range (IQR). Psychometric reliability was measured by the values of internal consistency of the items in the domain and the entire questionnaire presented by Cronbach’s alpha and strength of association between items and summary score of domains as well as the total score of the ACSS, presented by Pearson’s *r*-coefficient of product-moment correlation. Diagnostic values of the domains and items of the ACSS and diagnostic modalities were assessed by calculation of sensitivity, specificity, positive and negative likelihood ratios, diagnostic odds ratio (DOR), and ROC-curve analysis.

Comparative analysis was performed via paired *t*-test (for numerical data), with the Welch correction in cases of inequality of variances, Wilcoxon signed ranks test (for ordinal and interval data), and McNemar’s chi-square test (for categorical variables). Pearson’s correlation coefficient was used to assess the strength of associations between variables and the outcome, and the interval nature of the outcome variable (e.g., grouped number of CFU of pathogens in urine culture). Statistical significance was set at 0.05.

R v.3.5.2 with in-built and additional packages was used for the statistical analysis and graphical representation of the results [27,28,29,30].

## 5. Conclusions

The American English ACSS showed high values of predictive ability and responsiveness, and excellent levels of reliability and validity for diagnostics of acute UC and as a PRO measure. Therefore the ACSS can now be recommended as a new master version for clinical or epidemiological studies, but also in clinical practice and for self-diagnosis for women with symptoms of acute UC with American English as their first language.

## Figures and Tables

**Table 1:** Demographics and additional conditions (according to ACSS) of the US and international group at D1 (visit 1).

	US Study Group	International Study Group	
Patients	N (%)	N (%)	*p*^1^-value
Total	167 (100%)	237 (100%)	
Range age (years)	17–87	17–87	0.072
Mean age (SD)	36.8 (15.3)	34.6 (15.1)	
Median age (IQR)	32 (25;46)	30 (23;40)	
18–32 years	86 (51.5%)	138 (58.2%)	0.233
33–47 years	43 (25.8%)	50 (21.1%)	
48–62 years	21 (12.6%)	28 (11.8%)	
>62 years	17 (10.2%)	19 (8.0%)	
NA	-	2 (0.8%)	
			*p*^2^-value
Menstruation	19 (11.4%)	25 (10.6%)	0.943
PMS	8 (4.8%)	20 (8.4%	0.201
Menopause	11 (6.6%)	19 (8.0%)	0.676
Pregnancy	0 (0%)	27 (11.4%)	<0.001
Diabetes mellitus	2 (1.2%)	2 (0.8%)	1.000

SD, standard deviation; IQR, interquartile range; PMS, premenstrual syndrome. *p*^1^, Wilcoxon–Mann–Whitney test; *p*^2^, chi-square test.

**Table 2:** ACSS data derived from patients of the US group at Day 1 (diagnostics before the start of treatment).

ACSS		Total Patients (N = 167; 100%)	Symptom Severity (N, %)				
**Do-** **main**	Q	Symptoms	0 None (N, %)	1 Mild (N, %)	2 Moderate (N, %)	3 Severe (N, %)	2 + 3 Mod + Sev (N, %)
**Typical Symptoms**	1	Urinary frequency	3 (1.80%)	18 (10.78%)	82 (49.10%)	64 (38.32%)	146 (87.42%)
	2	Urinary urgency	1 (0.60%)	16 (9.58%)	90 (53.89%)	60 (35.93%)	150 (89.82%)
	3	Dysuria (burning pain when urinating)	12 (7.19%)	22 (13.17%)	76 (45.51%)	57 (34.13%)	133 (79.64%)
	4	Incomplete bladder emptying	3 (1.80%)	17 (10.18%)	96 (57.49%)	51 (30.54%)	147 (88.03%)
	5	Pain in lower abdomen (suprapubic pain)	46 (27.54%)	30 (17.96%)	72 (43.11%)	19 (11.38%)	91 (54.49%)
	6	Visible blood in urine	125 (74.85%)	20 (11.98%)	10 (5.99%)	12 (7.19%)	22 (13.18%)
**Differential Symptoms**	7	Flank pain	86 (51.50%)	30 (17.96%)	38 (22.75%)	13 (7.78%)	51 (30.53%)
	8	Abnormal vaginal discharge	106 (63.47%)	38 (22.75%)	18 (10.78%)	5 (2.99%)	23 (13.77%)
	9	Discharge from the urethra	125 (74.85%)	30 (17.96%)	12 (7.19%)	0 (0.00%)	12 (7.19%)
	10	High body temperature/fever	157 (94.01%)	9 (5.39%)	1 (0.60%)	0 (0.00%)	1 (0.60%)
**Quality of Life**	11	Discomfort because of symptoms	9 (5.39%)	27 (16.17%)	83 (49.70%)	48 (28.74%)	131 (78.44%)
	12	Interference with everyday activities/work	14 (8.38%)	47 (28.14%)	72 (43.11%)	34 (20.36%)	106 (63.47%)
	13	Interference with social activities	26 (15.57%)	43 (25.75%)	70 (41.92%)	28 (16.77%)	98 (58.69%)

Q = Questions of the ACSS.

**Table 3:** ACSS sum scores of typical symptoms, differential symptoms, and quality of life derived from the US and international group on Day 1.

	US Group		International Group		
ACSS	Patients	Sum Score	Patients	Sum Score	
Domain	N (Total)	Mean (SD)	N (Total)	Mean (SD)	*p*-Value *
Typical	167	10.60 (2.51)	237	10.12 (3.76)	0.155
Differential	167	1.79 (1.81)	237	2.39 (2.05)	0.003
QoL	167	5.37 (2.34)	237	5.58 (1.92)	0.443
ACSS total	167	17.72 (5.0)	237	18.08 (5.99)	0.314

* Student *t*-test.

**Table 4:** ACSS sum scores of (i) typical symptoms in relation to amount of bacteriuria, (ii) differential symptoms, and (iii) quality of life derived from the same patients of the US group at Day 1 (Diagnostics) and Day 5/6 (End of Therapy). *p*-paired *t*-test.

		Day 1 (Diagnostics)			Day 5/6 (End of Therapy)			
ACSS		Patients	Sum Score		Patients	Sum Score		
Domain		N (total)	Mean (SD)	Median (IQR)	N (total)	Mean (SD)	Median (IQR)	*p*-value
Typical	Total	100	10.49 (2.60)	10 (9;12)	100	0.98 (1.94)	0 (0;1)	<0.001
	<10^4^ CFU/mL	43	10.53 (2.59)	11 (9;12.5)	43	1.49 (2.60)	0 (0;2)	<0.001
	10^4^ CFU/mL	29	10.66 (2.93)	11 (9;13)	29	0.48 (1.12)	0 (0:0)	<0.001
	>10^5^ CFU/mL	28	10.25 (2.29)	10 (9;12)	28	0.71 (1.12)	0 (0;1.3)	<0.001
Differential		100	1.57 (1.74)	1 (0;3)	100	0.34 (0.62)	0 (0;1)	<0.001
QoL		100	5.48 (2.46)	6 (4;7)	100	0.46 (0.93)	0 (0;0)	<0.001
ACSS total		100	17.54 (5.30)	17 (14;22)	100	1.78 (2.82)	0 (0;2)	<0.001

**Table 5:** ACSS data derived from patients of the US group at Day 1 (diagnostics before the start of treatment) and Day 5/6 (end of treatment).

		Day 1 (Diagnostics)						Day 5/6 (End of Therapy)							0 + 1 vs. 2 + 3
ACSS		Total	Symptom Severity					Total	Symptom Severity						
**Do-** **main**	Q	N (100%)	0 None (N,%)	1 Mild (N,%)	2 Moderate (N,%)	3 Severe (N,%)	2 + 3 Mod/Sev (N,%)	N (100%)	0 None (N,%)	1 Mild (N,%)	2 Moderate (N,%)	3 Severe (N,%)	2 + 3 Mod/Sev (N,%)	*p*^1^-value	*p*^2^-value
**Typical** **Symptoms**	1	100	2	14	48	36	84	100	75	17	6	2	8	<0.001	<0.001
	2	100	0	10	55	35	90	100	79	19	1	1	2	<0.001	<0.001
	3	100	6	15	44	35	79	100	88	11	0	1	1	<0.001	<0.001
	4	100	3	9	54	34	88	100	90	9	0	1	1	<0.001	<0.001
	5	100	28	19	39	14	53	100	90	10	0	0	0	<0.001	<0.001
	6	100	77	11	7	5	12	100	98	1	1	0	1	<0.001	0.002
**Differential Symptoms**	7	100	54	21	18	7	25	200	85	13	2	0	2	<0.001	<0.001
	8	100	71	16	10	3	13	100	90	9	1	0	1	<0.001	0.001
	9	100	78	16	6	0	6	100	95	5	0	0	0	<0.001	0.014
	10	100	88	12	0	0	0	100	96	4	0	0	0	0.024	NA
**Quality** **of Life**	11	100	7	11	48	34	82	100	76	23	1	0	1	<0.001	<0.001
	12	100	10	26	41	23	64	100	89	11	0	0	0	<0.001	<0.001
	13	100	15	24	45	16	61	100	90	10	0	0	0	<0.001	<0.001
**Dyna-** **mics**									**Grading of “Dynamics“**						
									0	1	2	3	4		
								100	64	27	7	2	0		

Q, ACSS question; *p*^1^, Wilcoxon signed ranks test; *p*^2^, McNemar’s chi-square test.

**Table 6:** The number of cases using certain ACSS thresholds for clinical success at Day 5/6 in the patients of the US (n = 100) and international group at Day 5–9 (end of treatment) (n = 82) [10]. Note: each case with any “visible blood in the urine (VBU)” at end of treatment was rated “non-success”.

Type	Thresholds for Clinical Success	American English	International
A	Sum score of typical domain ≤5 scores, no item >1	91 (91%)	66 (80.49%)
B	Sum score of typical domain ≤5 scores, no item >1 and no item of QoL >1	91 (91%)	60 (73.17%)
C	Dynamics, no item >1	91 (91%)	64 (78.05%)
D	Sum score of the 4 FDA symptoms ≤4, no item >1	91 (91%)	66 (80.49%)
E	Sum score of the 3 EMA symptoms ≤3, no item>1	91 (91%)	67 (81.71%)

4 FDA symptoms (urinary frequency, urinary urgency, dysuria, suprapubic pain). 3 EMA symptoms (urinary frequency, urinary urgency, dysuria).

**Table 7:** Sensitivity, specificity, positive and negative predictive values, positive and negative likelihood ratio, and correlation with a positive outcome (PO for diagnosis of cystitis) using different criteria (mean and 95% CI). CI, confidence interval; PPV, positive predictive value, NPV, negative predictive value; +LR, positive likelihood ratio; −LR, negative likelihood ratio; AUC, area under the curve; PO, positive outcome (diagnosis is correct). Main symptoms and 3 EMA symptoms: urinary frequency, urinary urgency, dysuria; 4 FDA symptoms: urinary frequency, urinary urgency, dysuria, suprapubic pain.

Criteria	N (%) Positive	N (%) Negative	Sensitivity	Specificity	PPV	NPV	+LR	−LR	AUC	Correlation with PO
ACSS: typical domain, sumscore ≥6	96 (96%)	2 (2%)	0.96 (0.90–0.99)	0.98 (0.93–1.00)	0.98 (0.92–1.00)	0.96 (0.90–0.99)	48.00 (12.17–189.38)	0.04 (0.02–0.11)	0.97 (0.95–0.99)	0.94 (0.92–0.95)
ACSS: main symptoms, sumscore ≥6	77 (77%)	1 (1%)	0.77 (0.68–0.85)	0.99 (0.95–1.00)	0.99 (0.93–1.00)	0.81 (0.73–0.88)	77.00 (10.92–542.88)	0.23 (0.16–0.33)	0.88 (0.84–0.92)	0.78 (0.72–0.83)
FDA: at least 2 positive symptom of 4	100 (100%)	33 (33%)	1.00 (0.96–1.00)	0.67 (0.57–0.76)	0.75 (0.67–0.82)	1.00 (0.95–1.00)	3.03 (2.29–4.01)	0.00 (0.00-NA)	0.84 (0.79–0.88)	0.71 (0.63–0.77)
EMA: at least 1 positive symptom of 3	100 (100%)	22 (22%)	1.00 (0.96–1.00)	0.78 (0.69–0.86)	0.82 (0.74–0.88)	1.00 (0.95–1.00)	4.55 (3.14–6.57)	0.00 (0.00-NA)	0.89 (0.85–0.93)	0.80 (0.74–0.85)

## Supplementary Materials

The following are available online at https://www.mdpi.com/2079-6382/9/12/929/s1, Figure S1. Boxplots (IQR, range, mean ± SD) of the numbers of the ACSS typical symptoms in the US and in the international group. Figure S2. Boxplots (IQR, range, mean ± SD) of the summary scores of the ACSS typical symptoms in the US and in the international group. Figure S3: Boxplots (IQR, range, mean ± SD) of the severity scores of the six ACSS typical symptoms in the US group versus the three categories of bacteriuria at Day 1.

## Copyright of the ACSS

The ACSS is copyrighted by the Certificate of Deposit of Intellectual Property in Fundamental Library of Academy of Sciences of the Republic of Uzbekistan, Tashkent (Registration number 2463; 26 August 2015) and the Certificate of the International Online Copyright Office, European Depository, Berlin, Germany (Nr. EU-01-000764; 21 October 2015). The rightsholders are Jakhongir Fatikhovich Alidjanov (Uzbekistan), Ozoda Takhirovna Alidjanova (Uzbekistan), Adrian Martin Erich Pilatz (Germany), Kurt Guenther Naber (Germany), and Florian Martin Erich Wagenlehner (Germany). The e-USQOLAT is copyrighted by the Authorship Certificate of the International Online Copyright Office, European Depository, Berlin, Germany (Nr. EC-01-001179; 18 May 2017) 19. Translations of the ACSS in other languages are available on the website: http://www.acss.world/downloads.html.

## Abbreviations

ACSS	acute cystitis symptom score
AUC	area under the curve;
CFU	colony-forming units
CI	confidence interval
DOR	diagnostic odds ratio
EMA	European Medical Agency
EoT	end of treatment
FDA	Food and Drug Administration
IQR	interquartile range
+LR	positive likelihood ratio
-LR	negative likelihood ratio
N	number
NPV	negative predictive value;
PMS	premenstrual syndrome
PO	positive outcome (diagnosis is correct)
PPV	positive predictive value,
PRO	patient-reported outcome
*p*-value	probability value
Q	question of the ACSS
QID	four times a day
QoL	quality of life
ROC	Receiver operating characteristic
SC	scientific committee
SD	standard deviation
UC	uncomplicated cystitis
US	United States of America
UTI	urinary tract infection
VBU	visible blood in urine

## References

[1] Colgan R., Williams M. (2011). Diagnosis and treatment of acute uncomplicated cystitis. Am. Fam. Physician.

[2] Desforges J.F., Stamm W.E., Hooton T.M. (1993). Management of urinary tract infections in adults. N. Engl. J. Med..

[3] Barry H.C., Ebell M.H., Hickner J. (1997). Evaluation of suspected urinary tract infection in ambulatory women: A cost-utility analysis of office-based strategies. J. Fam. Pr..

[4] Colgan R., Keating K., Dougouih M. (2004). Survey of symptom burden in women with uncomplicated urinary tract infections. Clin. Drug Investig..

[5] Clayson D., Wild D., Doll H., Keating K., Gondek K. (2005). Validation of a patient-administered questionnaire to measure the severity and bothersomeness of lower urinary tract symptoms in uncomplicated urinary tract infection (UTI): The UTI Symptom Assessment questionnaire. BJU Int..

[6] Wild D., Clayson D.J., Keating K.N., Gondek K. (2005). Validation of a patient-administered questionnaire to measure the activity impairment experienced by women with uncomplicated urinary tract infection: The Activity Impairment Assessment (AIA). Health Qual. Life Outcomes.

[7] Alidjanov J.F., Naber K.G., Abdufattaev U.A., Pilatz A., Wagenlehner F.M. (2018). Reliability of Symptom-Based Diagnosis of Uncomplicated Cystitis. Urol. Int..

[8] Alidjanov J.F., Abdufattaev U.A., Makhsudov S.A., Pilatz A., Akilov F.A., Naber K.G., Wagenlehner F.M. (2014). New self-reporting questionnaire to assess urinary tract infections and differential diagnosis: Acute cystitis symptom score. Urol Int..

[9] Alidjanov J.F., Naber K.G., Pilatz A., Radzhabov A., Zamuddinov M., Magyar A., Tenke P., Wagenlehner F.M. (2020). Evaluation of the draft guidelines proposed by EMA and FDA for the clinical diagnosis of acute uncomplicated cystitis in women. World J. Urol..

[10] Alidjanov J.F., Naber K.G., Pilatz A., Radzhabov A., Zamuddinov M., Magyar A., Tenke P., Wagenlehner F.M. (2020). Additional assessment of Acute Cystitis Symptom Score questionnaire for patient-reported outcome measure in female patients with acute uncomplicated cystitis: Part II. World J. Urol..

[11] Food Drug Administration Center for Drugs Evaluation Research (2019). Uncomplicated Urinary Tract Infections: Developing Drugs for Treatment: Guidance for Industry.

[12] European Medicines Agency Committee for Human Medicinal Products (2018). Evaluation of medicinal products indicated for treatment of bacterial infections. Draft Guideline.

[13] Alidjanov J.F., Naber K.G., Pilatz A., Hurley M., Mohoney C.M., Barnes B.D., Werchan P., Wagenlehner F. (2018). The American English Acute Cystitis Symptom Score—Linguistic Validation. Proceedings of the 33rd Annual Congress of the European Association of Urology.

[14] Alidjanov J.F., Abdufattaev U.A., Makhmudov D., Mirkhamidov D., Khadzhikhanov F.A., Azgamov A.V., Pilatz A., Naber K., Wagenlehner F.M., Akilov F.A. (2014). Development and clinical testing of the Russian version of the Acute Cystitis Symptom Score—ACSS. Urologiia.

[15] Alidjanov J.F., Pilatz A., Abdufattaev U.A., Wiltink J., Weidner W., Naber K.G., Wagenlehner F. (2015). German validation of the Acute Cystitis Symptom Score. Urologe A.

[16] Alidjanov J.F. (2017). Preliminary Clinical Validation of the UK English Version of the Acute Cystitis Symptom Score in UK English-speaking female population of Newcastle, Great Britain. JOJ Urol. Nephrol..

[17] Di Vico T., Morganti R., Cai T., Naber K.G., Wagenlehner F.M., Pilatz A., Alidjanov J.F., Morelli G., Bartoletti R. (2020). Acute Cystitis Symptom Score (ACSS): Clinical Validation of the Italian Version. Antibiotics.

[18] Magyar A., Alidjanov J.F., Pilatz A., Nagy K., Arthanareeswaran V.K.A., Póth S., Bécsi A., Wagenlehner F.M., Naber K.G., Tenke P. (2017). The role of the Acute Cystitis Symptom Score questionnaire for research and antimicrobial stewardship. Validation of the Hungarian version. Central Eur. J. Urol..

[19] Heytens S., De Sutter A., Coorevits L., Cools P., Boelens J., Van Simaey L., Christiaens T., Vaneechoutte M., Claeys G. (2017). Women with symptoms of a urinary tract infection but a negative urine culture: PCR-based quantification of Escherichia coli suggests infection in most cases. Clin. Microbiol. Infect..

[20] Hooton T.M., Roberts P.L., Cox M.E., Stapleton A.E. (2013). Voided midstream urine culture and acute cystitis in premenopausal women. N. Engl. J. Med..

[21] Stamm W.E., Counts G.W., Running K.R., Fihn S., Turck M., Holmes K.K. (1982). Diagnosis of coliform infection in acutely dysuric women. N. Engl. J. Med..

[22] Alidjanov J.F., Naber K.G., Abdufattaev U.A., Pilatz A., Wagenlehner F.M. (2018). Reevaluation of the Acute Cystitis Symptom Score, a Self-Reporting Questionnaire. Part II. Patient-Reported Outcome Assessment. Antibioics.

[23] Alidjanov J.F., Naber K.G., Abdufattaev U.A., Pilatz A., Wagenlehner F.M.E. (2018). Reevaluation of the Acute Cystitis Symptom Score, a Self-Reporting Questionnaire. Part I Development, Diagnosis and Differential Diagnosis. Antibiotics.

[24] Acquadro C. (2004). Linguistic Validation Manual for Patient-Reported Outcomes (PRO) Instruments.

[25] Acquadro C., Conway K., Giroudet C., Mear I. (2012). Linguistic Validation Manual for Health Outcome Assessments.

[26] Acquadro C., Jambon B.D.E., Marquis P., Spilker B. (1996). Language and translation issues. Quality of Life and Pharmacoeconomics in Clinical Trials.

[27] R Core Team (2017). R: A Language and Environment for Statistical Computing.

[28] Robin X.A., Turck N., Hainard A., Tiberti N., Lisacek F., Sanchez J.-C., Müller M. (2011). pROC: An open-source package for R and S+ to analyze and compare ROC curves. BMC Bioinform..

[29] Stevenson M., Nunes T., Heuer C., Marshall J., Sanchez J., Thornton R., Reiczigel J., Robison-Cox J., Sebastiani P., Solymos P. (2017). epiR: Tools for the Analysis of Epidemiological Data. https://cran.r-project.org/web/packages/epiR/index.html.

[30] Wickham H. (2017). Tidyverse: Easily Install and Load the ‘Tidyverse’. https://cran.r-project.org/web/packages/tidyverse/index.html.

