# Supplementary figures and images for: Validation of the American English Acute Cystitis Symptom Score
Source: Antibiotics (Basel). 2020 Dec 19;9(12):929. doi: 10.3390/antibiotics9120929 (PMC7766804; doi:10.3390/antibiotics9120929)

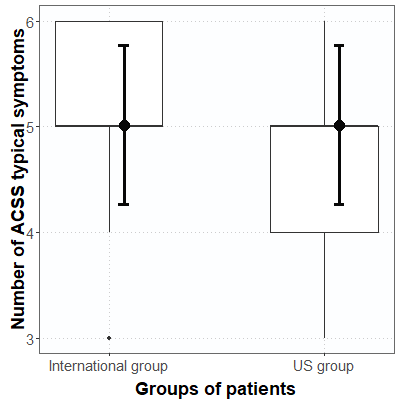

Supplement: Supplementary file 1 [file antibiotics-09-00929-s001.zip › suppl.fig.1.boxplots.tif]

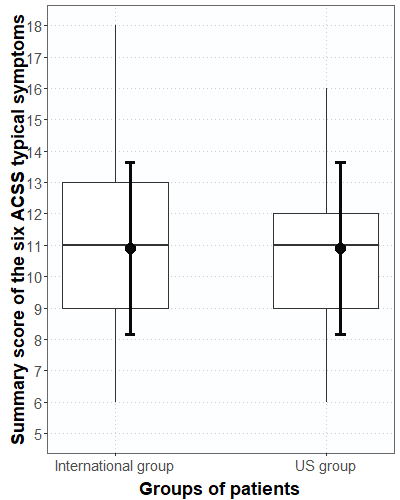

Supplement: Supplementary file 1 [file antibiotics-09-00929-s001.zip › suppl.fig.2.boxplots.tif]

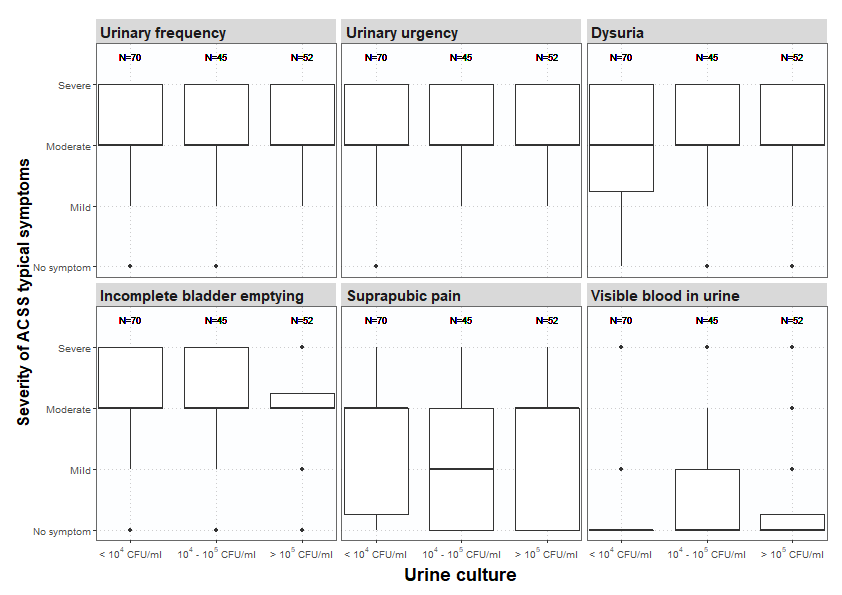

Supplement: Supplementary file 1 [file antibiotics-09-00929-s001.zip › suppl.fig.3.boxplots.tif]
